# ITF2357 transactivates Id3 and regulate TGFβ/BMP7 signaling pathways to attenuate corneal fibrosis

**DOI:** 10.1038/srep20841

**Published:** 2016-02-11

**Authors:** Rayne R. Lim, Alison Tan, Yu-Chi Liu, Veluchamy A. Barathi, Rajiv R. Mohan, Jodhbir S. Mehta, Shyam S. Chaurasia

**Affiliations:** 1 Ocular Immunology and Angiogenesis Lab, Department of Veterinary Medicine & Surgery, University of Missouri, Columbia, MO 65211, USA; 2 Department of Biomedical Sciences, University of Missouri, Columbia, MO 65211, USA; 3 Singapore Eye Research Institute, 20 College Road, 169856, Singapore; 4 Institute of Molecular and Cellular Biology, A*Star, Biopolis, Singapore; 5 Singapore National Eye Centre, 11 Third Hospital Avenue, 168751, Singapore; 6 Ophthalmology and Visual Sciences Academic Clinical Program, Duke-NUS Graduate Medical School, 8 College Rd, 169857, Singapore; 7 Department of Ophthalmology, Yong Loo Lin School of Medicine, National University of Singapore, Singapore; 8 Harry S. Truman Memorial Veteran Hospital, Columbia, MO 65201, USA; 9 Mason Eye Institute, University of Missouri, Columbia, MO 65211, USA

## Abstract

Corneal fibrosis is often seen in patients with ocular trauma and infection that compromises corneal transparency resulting in vision loss. Treatment strategies including NSAIDs, steroids, MMC and corneal transplants have shown tremendous success but with several side effects and cellular toxicity. Histone deacetylase inhibitors (HDACi) have been shown to inhibit corneal fibrosis via TGFβ signaling pathway. In this study, we investigated safety, efficacy and mechanism of action of a HDACi, ITF2357 in TGFβ-stimulated *in vitro* primary human cornea stromal fibroblasts (pHCSFs) and *in vivo* in a photorefractive keratectomy-treated rabbit model of corneal fibrosis. We found that *in vivo* ITF2357 decreased collagen I, collagen IV, fibronectin, integrin αVβ3 expression with a reduction in corneal haze. In addition, ITF2357 reduced myofibroblast formation, suppressed phosphorylation of Smad proteins in TGFβ pathway and inhibited key responsive protein, P4HA1 involved in pro-collagen synthesis. Treatment of pHCSFs with ITF2357 activated BMP7 levels and expressed all the members of inhibitor of differentiation proteins (Id1-Id4), however, it failed to rescue TGFβ-driven transdifferentiation of fibroblasts to myofibroblasts in the presence of siRNA specific to Id3. We conclude that ITF2357 is a potential anti-fibrotic drug that exerts its action via activation of Id3, a downstream target of TGFβ/BMP7 signaling pathways.

Cellular transdifferentiation of stromal fibroblast to myofibroblast is described as a critical rate-limiting step in the corneal wound healing mechanism^1^,^2^,^3^,^4^. These metabolically active and highly contractile cells are essential for effective wound closure through deposition of extracellular matrix (ECM) and reorganization of stromal collagen bed after injury. They are characterized by the stromal expression of α-smooth muscle actin (αSMA)^5^ and preceded by intermittent precursor cells expressing vimentin and desmin^6^. Post-injury corneal myofibroblasts usually undergo apoptosis and pave way to the infiltrating keratocytes to effectively close the wound^7^. However, pathological accumulation of myofibroblasts accompanied with the excessive secretion of irregular ECM matrix results in aberrant wound healing and hence corneal fibrosis. This is clinically recognized as corneal haze in patients, which may lead to loss of vision.

Current treatment strategies involve off-label use of Mitomycin C (MMC) in clinics, which has been controversial because of its dosage and long-term efficacy in patients^8^,^9^. Few reports have suggested it to be safe and effective in patients^10^ while long-term use of MMC has been associated with wound healing dysfunction^11^. Other treatment strategies such as steroidal intervention have been shown to be associated with raised intraocular pressure in some cases and possible cataract formation^12^. Surgical procedures such as phototherapeutic keratectomy (PTK) are often limited to superficial scarring^13^. Therefore, there is an unmet need for a safe and effective drug against corneal fibrosis.

Transforming growth factor-beta (TGFβ) secreted by the injured corneal epithelial cells play a predominant role in corneal wound healing^14^. Epithelial-to-mesenchymal transition (EMT) has been shown to produce myofibroblasts^15^ and overexpression of TGFβ can lead to unregulated ECM production, coupled with decreased ECM degradation. Neutralizing antibody to TGFβ has been reported to reduce myofibroblasts in post-PRK corneas^16^. TGFβ signals downstream effects via Smad signaling proteins^17^. In brief, Smad2 and Smad3 dimerizes as a result of phosphorylation by serine/threonine TGFβ receptor complexes, and translocate into the nucleus with the association of co-activator, Smad4. Inhibitory Smad7 antagonizes TGFβ pathway by interfering with Smad2/3 recruitment. Previous studies have targeted TGFβ and its signaling components for the inhibition of corneal haze^18^,^19^. A recent study by Wang *et al.*^20^ used lentiviral transfection of Smad7 to inhibit fibrogenic response in the rat cornea after PRK. Similarly, decorin, an endogenous TGFβ inhibitor, has been shown to suppress fibrogenesis in human corneal fibroblasts^21^ and inhibit corneal haze after targeted gene therapy in a rabbit model of corneal fibrosis^22^. In addition, gene delivery using BMP7 encapsulated nanoparticles has been demonstrated as potential treatment strategy for corneal fibrosis^23^. However, most of these results were obtained from end-point analysis and lack detailed molecular mechanisms responsible for their anti-fibrotic role. Since TGFβ signaling pathway is involved in diverse cellular processes, it will not be biologically pertinent to completely block the entire pathway.

Histone deacetylase inhibitors (HDACi) are an emerging class of drugs used in medicine due to transcriptional activation of DNA via histone acetylation. They are associated with the regulation of cell migration, cell differentiation, cell-cycle arrest and angiogenesis^24^. Their clinical uses have ranged from cancer^25^ and neurological conditions^26^, to autoimmune^27^ and autoinflammatory diseases^28^. Recent studies have suggested promising results on the anti-fibrotic role of HDAC inhibitors in pulmonary fibrosis^29^, cardiac hypertrophy^30^ and renal fibrosis^31^. In the cornea, HDACi have been reported to inhibit myofibroblast formation. First generation HDACi such as Trichostatin A (TSA)^32^,^33^ and its derivative Vorinostat (SAHA)^34^,^35^ both demonstrated their effectiveness in preventing fibrosis. However, the molecular mechanisms have not yet been elucidated.

ITF2357 (Givinostat), is a second generation synthetic pan-HDACi that belongs to the hydroxamic acids family with HDAC type I and type II inhibitory action^36^. Similar to other HDACi, ITF2357 has a potent anti-neoplastic activity on multiple myeloma^37^, acute myelogenous leukemia cells^38^ and non-small cell lung cancer^39^. Interestingly, ITF2357 has also been advocated for its anti-inflammatory functions in autoimmune disorders such as Crohn’s disease, juvenile idiopathic arthritis, and myeloproliferative diseases^40^,^41^,^42^. In humans, single or repeated oral administration of ITF2357 (50–100 mg/day) was found to be safe with ~5–7 hr half-life and played a vital role in the inhibition of circulating inflammatory cytokines^43^. In particular, ITF2357 has been suggested to be active and anti-inflammatory in low nanomolar concentrations^44^,^45^ as opposed to TSA. On the other hand, SAHA was found to be ineffective at low concentrations^44^,^46^ and hyperacetylated histones in human peripheral blood mononuclear cells (PBMCs) for only up to 6 hr compared to ITF2357 hyperacetylation that sustained for more than 24 hr^36^. In addition, anti-inflammatory potency of ITF2357 was found to be 25–50 fold more effective than SAHA *in vitro* and *in vivo*^45^,^46^,^47^. However, the anti-fibrotic role of ITF2357 has not yet been studied.

In the present study, we investigated the safety and efficacy of ITF2357 in abrogating fibrosis *in vitro* using TGFβ-stimulated primary cultures of human corneal fibroblasts (pHCSFs), and *in vivo* in a photorefractive keratectomy (PRK)-treated rabbit model of corneal fibrosis. In addition, we also elucidated molecular mechanisms underlying the anti-fibrotic role of ITF2357 in the regulation of TGFβ/Smad/BMP7 signaling axis and downstream targets involved in the inhibition of corneal fibrosis.

## Results

### ITF2357 is safe and effective at nanomolar concentrations in pHCSFs

**Figure 1:**
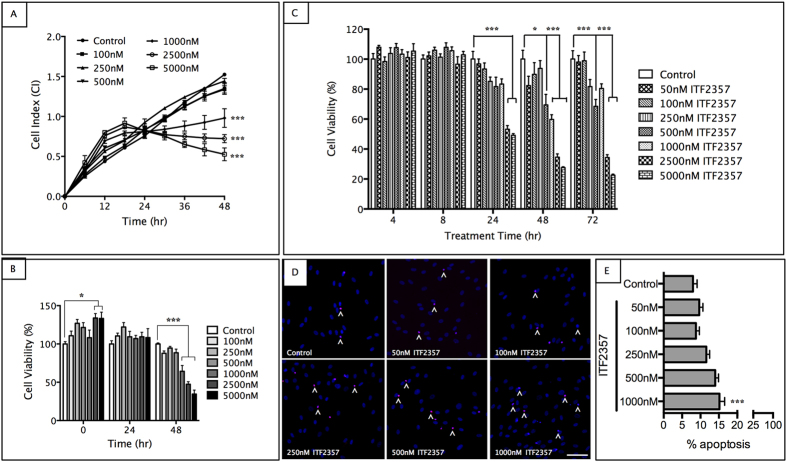
ITF2357 is safe in nanomolar concentrations. (**A**) xCELLigence assay showed no significant decrease in cell index for primary human corneal stromal fibroblasts (pHCSFs) treated with 100–500 nM ITF2357, and a graded reduction in cultures treated with 1000–5000 nM ITF2357 for 48 hr (n = 6). (**B**) Time-point analysis of cell index from xCELLigence assay reflected decreased cell viability in pHCSFs treated (n = 6) at higher concentrations of ITF2357 (≥1000 nM). (**C**) PrestoBlue assay showed similar cell survival profile with concentrations of ≥1000 nM ITF2357 being toxic to pHCSFs treated for ≥48 hr (n = 6). (**D,E**) TUNEL assay indicated a significant increase in apoptotic cells (^) at ITF2357 concentrations above 1000 nM after 72 hr treatment (n = 11–17). Scale bar = 50μm. *p < 0.05 and ***p < 0.001 against control group. Error bars represent SEM.

To study the safety profile of ITF2357, primary cultures of human corneal stromal fibroblasts (pHCSFs) were established from human donor corneal rims in DMEM/F-12 medium containing 10% FBS, 1X insulin-transferrin-selenium, 1% penicillin/ streptomycin and 0.1% amphotericin B. We used an impedance-based real time monitoring of cell growth, cell shape and cell death over a period of 48 hr using xCELLigence RTCA system. Cells were seeded into a 96 well E-plate, starved for 24 hr and treated with varying concentrations of ITF2357 ranging from 100–5000 nM concentrations. ITF2357-treated cells showed time-dependent increase in growth and proliferation as measured by cell index (CI; Fig. 1A,B). pHCSFs growth plateaued in 1000–2500 nM concentrations with CI values of 0.8 after 24 hr, which was sustained until 48 hr, indicating the lack of both cell growth and cell death at these concentrations. However, pHCSFs with 5000 nM ITF2357 treatment showed a clear drop in CI values from 0.8 to 0.5 (Fig. 1A). CI was further evaluated by time-point analysis, where CI of all pHCSFs treated with ITF2357 was normalized against the CI of control pHCSFs at each time point (Fig. 1B). Results showed similar profile where ITF2357 in concentrations <1000 nM was safe on pHCSFs cell growth. However, concentrations above 2500 nM reflected a reduction in cell viability up to 50% compared to control at 48 hr. These results were confirmed with resazurin-based PrestoBlue® cell viability assay, which allows the repeated monitoring of cells over a period of time. The absorbance values of ITF2357-treated groups were normalized to the control group at each time point analyzed. pHCSFs showed ~50% reduction in cell viability with ITF2357 concentrations >1000 nM at 48 hr, which was further reduced to ~20% in the 5000 nM treated group at 72 hr (Fig. 1C).

TUNEL assay was employed to further evaluate the safety of the drug. ITF2357 at 50–1000 nM concentrations showed dose dependent increase in the apoptotic cells after 72 hr treatment. pHCSFs exhibited 10–15% overall cell death with >500 nM ITF2357, and showed 2-fold increase (p < 0.001) with 1000 nM ITF2357 as compared to control group (Fig. 1D,E).

### ITF2357 regulates pHCSFs cell migration and wound closure in a dose-dependent manner

Scratch wound assay performed on confluent pHCSFs showed steady migration of fibroblasts into the scratched area (Supplementary figure S1) and closed the wound within 48–96 hr. ITF2357 at concentrations <500 nM showed no differences in cell migration pattern, while significant retardation on migration was seen in 1000 nM ITF2357 group, but only after 96 hr of treatment (p < 0.001). This retarded cell movement in 1000 nM ITF2357 treated group can be attributed to reduced cell proliferation and growth, as minimal cell death was observed, corroborating to the overall 5–10% apoptosis seen in TUNEL assay. However, we found that ITF2357 at concentrations above 2500 nM caused significant cell death by 48 hr.

### ITF2357 hyperacetylates nuclear histone proteins extracted from pHCSFs

HDACi are known to hyperacetylate nuclear histone proteins, hence we examined whether ITF2357 produced similar modifications in pHCSFs. Nuclear proteins were extracted from pHCSFs treated with ITF2357, and analyzed by ac-lysine antibody using western blots. We observed a dose dependent increase in acetylation on lysine residues of histone proteins extracted from pHCSFs after treatment with ITF2357 (Supplementary figure S2).

### ITF2357 inhibits myofibroblast formation and irregular extracellular matrix (ECM) proteins in pHCSFs

**Figure 2:**
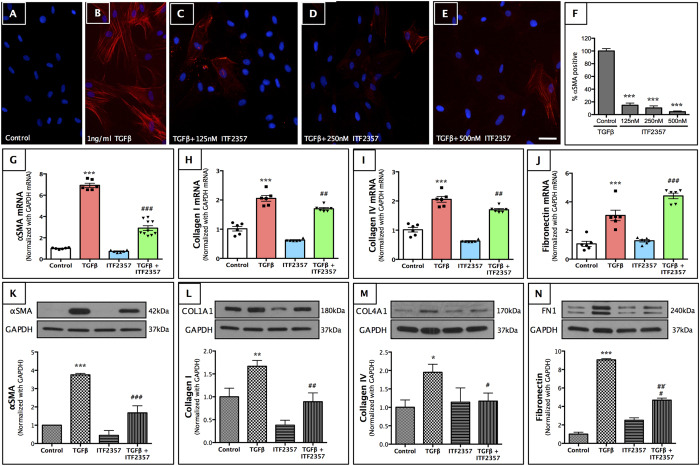
ITF2357 inhibits corneal fibrosis in pHCSFs at nanomolar concentrations. (**A–E**) αSMA immunostaining showed myofibroblast formation in pHCSFs treated with 1 ng/ml TGFβ (**B**). ITF2357 treatments at (**C**) 125 nM, (**D**) 250 nM or (**E)** 500 nM concentrations attenuated formation of myofibroblasts (Scale bar = 50 μm). (**F**) αSMA-positive cell quantification (n = 6–12) showed at least 80% down-regulation of αSMA staining in ITF2357 treated groups compared to TGFβ treated group (***p < 0.001). (**G**) αSMA mRNA upregulated 6-fold with TGFβ, which was decreased to only 3-fold in the presence of 250 nM ITF2357. (**K**) αSMA protein expression was similarly downregulated with 250 nM ITF2357 drug treatment. (**H**) Collagen I mRNA expression was increased in TGFβ group while significantly downregulated in the presence of 250 nM ITF2357. (**L**) This was also translated to protein COL1A1 expression with the same pattern. (**I**) Collagen IV mRNA production was similar to that of collagen I profile, with high transcript and (**M**) protein levels with TGFβ, which was significantly decreased in the presence of ITF2357. (**J**) In contrast fibronectin depicted increased mRNA in the presence of ITF2357 and TGFβ, which was not translated in (**N**) protein levels. **p < 0.01 and ***p < 0.001 against control; ^#^p < 0.05; ^##^p < 0.01 and ^###^p < 0.001 against TGFβ. There were n = 6 samples in each group and error bars represent SEM.

To determine the anti-fibrotic effects of ITF2357 at the cellular level, we used the classical *in vitro* model of corneal fibrosis where pHCSFs were stimulated with 1 ng/ml TGFβ. In brief, cells were treated with 125, 250 and 500 nM of ITF2357 in the presence of TGFβ, and fibroblasts to myofibroblasts transdifferentiation was evaluated by αSMA immunofluorescence. We observed a dose-dependent reduction in αSMA expression (Fig. 2A–F, p <0.001 compared to control in all the groups) at the transcript (Fig. 2G, p < 0.001 compared to the TGFβ treated group) and protein levels (Fig. 2K, p < 0.001 compared to the TGFβ treated group). Taking these results together with the safety profile data obtained earlier, 250 nM of ITF2357 was chosen as the optimal concentration for subsequent experiments on pHCSFs. Since myofibroblasts are known to secrete irregular matrix, we further evaluated the effect of ITF2357 on ECM at the transcript and protein levels. ITF2357 treatment showed significant down-regulation of collagen I (Fig. 2H, p < 0.01 for mRNA and Fig. 2L, p < 0.01 for protein) and collagen IV (Fig. 2I,M, p <0.01 for both mRNA and protein, respectively) compared to the TGFβ treated group. Fibronectin showed an unusual increase at the transcript levels with TGFβ in the presence of ITF2357 (Fig. 2J, p < 0.001), which was not translated into protein (Fig. 2N). In fact, there was a significant decrease in the fibronectin protein in ITF2357 + TGFβ treated group (Fig. 2N, p < 0.001). These results confirm the anti-fibrotic effects of ITF2357 upon TGFβ stimulation in pHCSFs.

### ITF2357 decreases anterior stromal reflectivity and reduces clinical haze in a rabbit model of corneal fibrosis

Since ITF2357 showed anti-fibrotic effects at the cellular level, we wanted to examine its functionality in an *in vivo* rabbit corneal fibrosis model established previously^6^. To test this, rabbit corneas underwent −9.0 diopter (−9.0D) photorefractive keratectomy (PRK) surgery, were treated with 0.02% ITF2357 and followed for 4 wk. MMC, which is currently used in several clinics served as a positive control. The experimental plan is illustrated in Supplementary figure S3. Both the drugs were applied topically immediately after PRK, and corneas were examined for haze formation weekly over a period of 4 wk.

**Figure 3:**
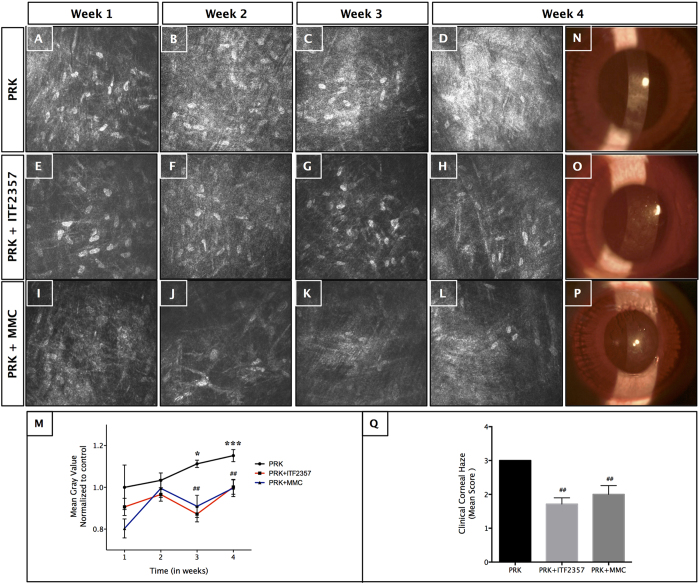
*In vivo* topical treatment of ITF2357 decreases anterior stromal reflectivity and reduced clinical haze. Confocal biomicroscopy images from wk 1 to 4 in (**A–D**) −9.0D photorefractive keratectomy (PRK), (E-H) PRK +0.2% ITF2357 and (**I–L**) PRK +0.02% MMC treated rabbits. (**D**) Maximum corneal haze was observed in wk 4, which was decreased by application of (H) ITF2357 and (**L**) MMC. (**I–L**) Fewer keratocytes were observed in the anterior stromal bed in MMC treated corneas. (**M**) Mean gray value analysis of confocal images showed maximum haze in PRK group at wk 3 and wk 4 (*p < 0.05 for wk 3 and ***p < 0.001 for wk 4 against control group). ITF2357 and MMC treatment significant reduced mean gray value by wk 3 and wk 4 (^##^p < 0.01 against PRK of wk 3 and wk 4 groups). Representative slit lamp microscopy images of corneal haze 4 wk after (**N**) PRK, (**O**) PRK + ITF2357 and (**P**) PRK + MMC rabbits showed similar reduction of clinical haze in MMC and ITF2357 treated corneas. (**Q**) Clinical grading of corneal haze over 4 wk post-PRK showed significant decrease in haze mean score after 1 month in both ITF2357 and MMC groups (^##^p < 0.01 against PRK group). There were six rabbits in each group and error bars represent SEM.

Confocal biomicroscopy of rabbit corneas that underwent PRK showed a progressive increase in anterior stromal reflectivity as a result of activated keratocytes and stromal disarrangement (Fig. 3A–D). Maximal haze was seen at wk 3 (Fig. 3C) and wk 4 (Fig. 3D), as quantified by measurement of mean gray value using Image J (Fig. 3M, p < 0.05 for wk 3 and p < 0.001 for wk 4 PRK groups compared to untreated controls). Rabbit corneas treated with 0.02% ITF2357 (Fig. 3E–H) or 0.02% MMC (Fig. 3I–L) showed significant reduction in reflectivity by wk 3 (Fig. 3M, p < 0.01 for both ITF2357 and MMC groups compared to PRK wk 3 group) that reached normal values observed in the control rabbits at the end of 4 wk (Fig. 3H–L for ITF2357 and MMC, p < 0.01 for both the groups compared to the PRK treated wk 4 samples). However, we observed a significant loss of keratocytes in the PRK corneas treated with MMC from wk 1–4 (Fig. 3I–L) compared to the ITF2357 treated group (Fig. 3E–H). Similar results were obtained with slit lamp microscopy (Fig. 3N–P), where we found a significant decrease in the clinical haze in ITF2357 (Fig. 3O) and MMC (Fig. 3P) treated post-PRK corneas by 4 wk. Clinical haze score calculated using grading method described by Fantes *et al.*^48^ also showed a significant inhibition of corneal haze by wk 4 compared to the control group (Fig. 3Q, p < 0.01 for both ITF2357 and MMC groups, respectively).

### ITF2357 is safe on corneal stroma

Corneal topography examined immediately following rabbit PRK and drug treatment showed similar pattern of central ablation (Supplementary figure S4C-D), and similar central cornea thickness was obtained over 4-wk follow up using ASOCT biomicroscopy (Supplementary figure S4A, B and E), indicating normal cornea recovery in ITF2357 or MMC treated groups. In addition, we also observed less reflectivity in the drug-treated groups compared to the PRK only group (data not shown). However, it was interesting to note that 0.02% ITF2357 was applied directly on the cornea compared to the MMC, which was applied by placing a soaked sponge directly on to the corneal ablation zone, followed by copious washing with balanced salt solution (BSS) as performed in the clinics.

### ITF2357 decreases *in vivo* corneal fibrosis and associated stress fibers

**Figure 4:**
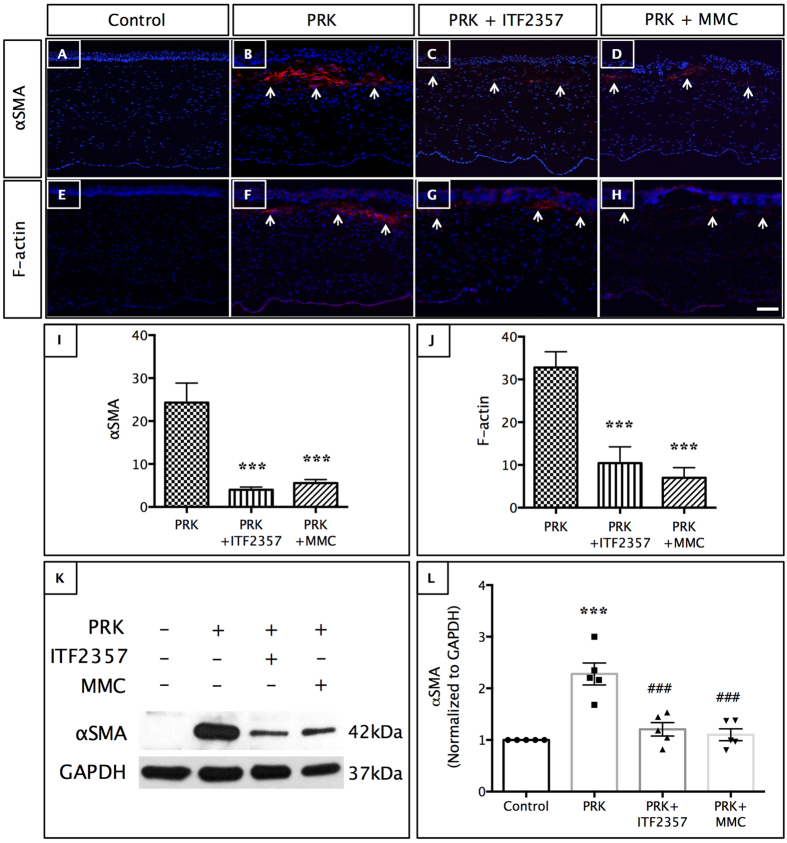
ITF2357 decreases myofibroblast formation. (**A–H**) Immunohistochemistry of (**A–D**) αSMA and (**E-H**) F-actin expression in rabbit cornea showed reduced post-PRK myofibroblast formation (↑) after (**C,G**) ITF2357 and (**D,H**) MMC treatment. Scale bar = 50 μm. (**I,J**) Significant decrease of ~80% was observed in αSMA-positive cells and F-actin-positive cells. ***p < 0.001 against PRK. (**K,L**) Western blot analysis with αSMA showed increased expression in PRK model, which was decreased in ITF2357 or MMC post-treated PRK corneas. ***p < 0.001 against control; ^###^p < 0.001 against PRK. There were n = 6 samples in each group and error bars represent SEM.

Next, we performed biochemical analysis on rabbit corneas to evaluate the role of ITF2357 on myofibroblasts and ECM formation in PRK treated corneas after 4 wk. In corneas treated with 0.02% ITF2357 or MMC, there was a significant reduction in the expression of myofibroblast marker, αSMA (Fig. 4A–D) as observed by immunohistochemistry. Quantification of αSMA positive cells showed ~80% reduction in myofibroblast formation (Fig. 4I, p < 0.001 in both ITF2357 and MMC treated PRK groups). Western blot analysis of corneal protein extracted after PRK treatment showed a significant increase in the αSMA expression (p < 0.001), which was rescued after treatment with ITF2357 or MMC treatment (Fig. 4K–L, p < 0.001 for both the drug treated groups compared to the TGFβ treated group).

F-actin is produced as stress fibers by myofibroblast during corneal wound repair, hence a reduction in activated keratocytes would result in a reduction of F-actin positive cells. In the present study, ITF2357 as well as MMC caused a significant decrease in F-actin expression in PRK corneas by immunohistochemistry (Fig. 4E–H) as well as quantitative analysis (Fig. 4J, p < 0.001 for both drug treated groups compared to the PRK treated corneas).

### ITF2357 downregulates irregular ECM proteins secreted by myofibroblasts

**Figure 5:**
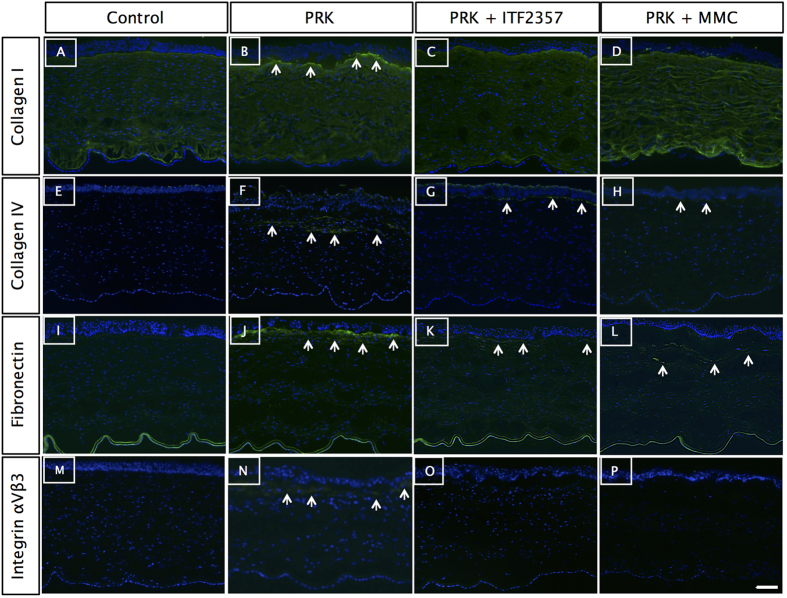
ITF2357 attenuates pro-fibrotic proteins. Extracellular matrix proteins (↑) (**A–D**) collagen I, (**E–H**) collagen IV, **(I–L**) fibronectin, (**M–P**) integrin αVβ3 were upregulated in (**B,F,J,N**) 4 wk post-PRK corneas. Collagen I and integrin αVβ3 staining was reduced in (**C,O**) ITF2357 and (**D**,**P**) MMC treated corneas. Similar reduction levels of collagen IV and fibronectin expression was observed in (**G,H,K,L**) post-PRK corneas treated with drugs. There were n = 4 samples in each group and scale bar = 50 μm.

Myofibroblasts are known to produce ECM matrix proteins for the remodeling of the stromal tissue during corneal wound healing^1^. Therefore, we investigated the role of ITF2357 treatment in the regulation of ECM proteins in PRK corneas. Immunohistochemistry of collagen I (Fig. 5A–D), collagen IV (Fig. 5E–H) and fibronectin (Fig. 5I–L) in 4 wk post PRK + ITF2357 or PRK + MMC treated corneas showed significant reduced levels of ECM proteins compared to the PRK treated corneas (Fig. 5). Integrin αVβ3, a transmembrane receptor that bridges cell-ECM interaction, has been reported to be essential for myofibroblast differentiation^49^. In this study, we found an increased expression of integrin αVβ3 after PRK surgery followed by decreased levels in ITF2357 or MMC-treated groups (Fig. 5M–P). It is interesting to note that the epithelial cell layers in the ITF2357 treated PRK corneas were more stratified at wk 4 compared to MMC treated corneas. In addition, there were more viable keratocytes observed in the anterior stroma in the area close to excimer laser ablation in the ITF2357 treated PRK corneas compared to the PRK or PRK + MMC groups.

### ITF2357 inhibited TGFβ/Smad signaling pathway

**Figure 6:**
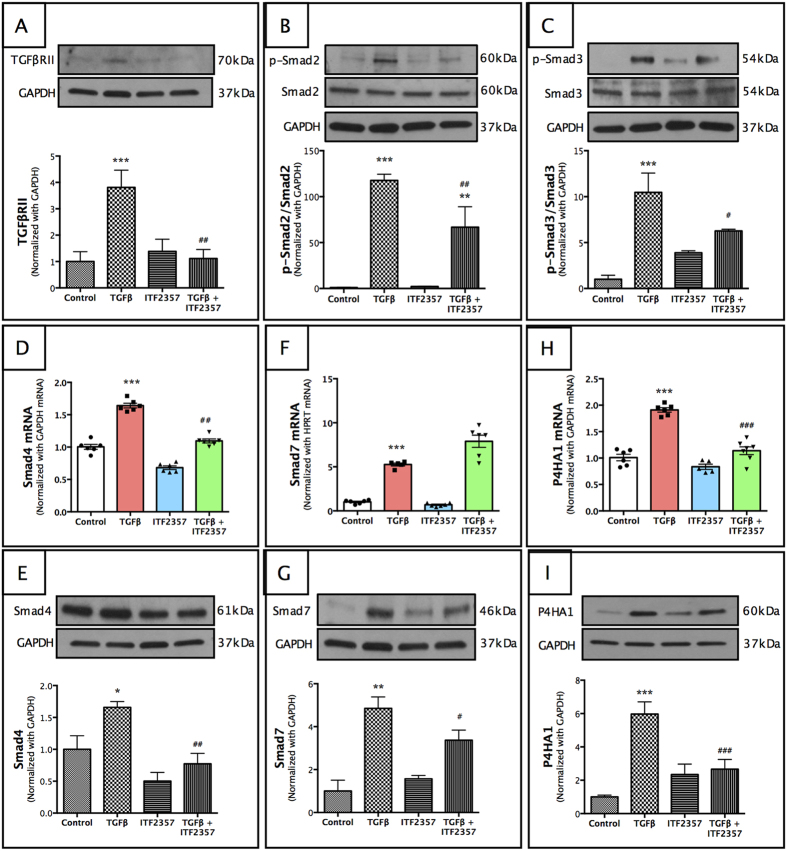
ITF2357 shows inhibitory effect on TGFβ/Smad signaling pathway. To elucidate mechanistic effects of ITF2357, key members of TGFβ/SMAD pathway were investigated. ITF2357 attenuated the TGFβ-induced expression of (**A**) TGFβRII protein and (**D**) Smad4 mRNA and (**E**) protein. (**F**) Smad7 mRNA was increased in TGFβ + ITF2357 group, but was reduced in (**G**) protein expression. Phosphorylation of (**B**) Smad2 and (**C**) Smad3 were both downregulated in the presence of ITF2357. (**H,I**) P4HA1 expression was increased with TGFβ treatment, which was significantly reduced with addition of 250 nM ITF2357. *p < 0.05; **p < 0.01 and ***p < 0.001 against control; ^#^p < 0.05; ^##^p < 0.01 and ^###^p < 0.001 against TGFβ. There were n = 6 samples for mRNA expression and n = 4 samples for protein expression in each group. Error bars represent SEM.

Since TGFβ is known to stimulate downstream fibrotic pathway via activation of Smads^17^, we investigated the role of ITF2357 in the regulation of key Smad members in pHCSFs (Fig. 6A–I). TGFβ treatment to pHCSFs increased expression of TGFβ type II receptor (TGFβRII) (Fig. 6A, p < 0.001 compared to the control group), which was antagonized by ITF2357 (p < 0.01 compared to the TGFβ group). Phosphorylated Smad2 and Smad3 binds to co-activator, Smad4 and translocates into the nucleus to activate transcription of TGFβ responsive genes. ITF2357 treatment to pHCSFs in the presence of TGFβ decreased phosphorylation of Smad2 (Fig. 6B, p < 0.01 compared to the TGFβ group) and Smad3 (Fig. 6C, p < 0.05 compared to the TGFβ group). At the same time, ITF2357 also inhibited the levels of co-activator Smad4 both at the transcript and protein levels (Fig. 6D–E, p < 0.01 for both the groups) compared to the TGFβ treatment. Smad7 is a target gene for Smad2/3-mediated transcription. We observed a marginal decrease in Smad7 protein (Fig. 6G, p < 0.05) but no changes in its transcript levels (Fig. 6F) suggesting an overall inhibitory activity of Smad2/3 signaling in the presence of ITF2357.

Most interestingly, pHCSFs treated with ITF2357 + TGFβ inhibited downstream TGFβ-responsive gene-prolyl 4-hydroxylase subunit α-1 (P4HA1), an enzyme responsible for maturation of pro-collagen to collagen fibers^50^, both at the transcript (Fig. 6H, p < 0.001) and protein levels (Fig. 6I, p < 0.001), counteracting the increased expression observed in the TGFβ treated group (Fig. 6H-I, p < 0.01 in both mRNA and protein levels compared to the control group). Thus, ITF2357 not only inhibited TGFβ signaling components but also decreased the activity of its downstream target, P4HA1.

### ITF2357 increases BMP7 production and activates BMP7/Smad signaling pathway

**Figure 7:**
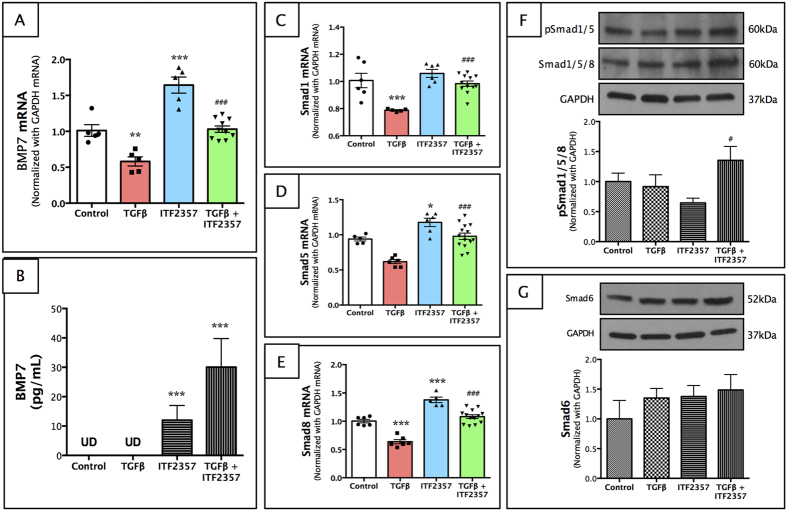
ITF2357 increases BMP7 production in BMP7/Smad signaling pathway. (**A**) BMP7 transcript was upregulated by ITF2357, but attenuated with TGFβ. (**B**) BMP7 protein was undetectable in the control and TGFβ as seen with ELISA assay, but showed a ~15-fold increase in the presence of ITF2357 and ~40-fold increase in the ITF2357 + TGFβ group. TGFβ stimulation to pHCSFs decreased (**C**) Smad1, (**D**) Smad5 and (**E**) Smad8 expression, which was restored by the treatment of ITF2357. However, (**F**) pSmad1/5/8 protein only showed a slight increase in the TGFβ + ITF2357 group. Moreover, we found no significant changes in (**G**) Smad6 protein in any of the group studied. *p < 0.05; **p < 0.01 and ***p < 0.001 against control; ^#^p < 0.05 and ^###^p < 0.001 against TGFβ. There were n = 6 samples for mRNA expression and ELISA assay whereas n = 4 for protein expression. Error bars represent SEM.

BMP7 is known to antagonize the TGFβ signaling pathway, and BMP7 gene therapy has been reported to inhibit corneal fibrosis *in vivo*^23^. Hence, we tested whether ITF2357 regulates BMP7 or its pathway components. ITF2357 activates BMP7 expression in pHCSFs with (Fig. 7A, p < 0.001 compared to TGFβ group only) or without TGFβ treatment (Fig. 7A, p < 0.001 compared to control). We also collected culture media from these cells and found a remarkable increase in the BMP7 levels in the ITF2357 treated groups as measured by ELISA assay (Fig. 7B, p < 0.001 for ITF2357 only and ITF2357 + TGFβ groups). In contrast, BMP7 was undetectable in control and/or TGFβ treated groups (Fig. 7B).

Next, we studied BMP7 pathway components-Smad1, Smad5 and Smad8 phosphorylation. While pHCSFs treated with TGFβ showed a significant decrease in Smad1 (Fig. 7C, p < 0.001), Smad3 (Fig. 7D, p < 0.001) and Smad8 (Fig. 7E, p < 0.001) expression compared to the control group, ITF2357 restored transcript levels to that of the control values (Fig. 7C–E, p < 0.001 compared to the TGFβ treated group). Western blot analysis showed a marginal increase in pSmad1/5 in the ITF2357 + TGFβ group (Fig. 7F, p < 0.05 compared to control group), whereas Smad6 protein remained unchanged in all the groups studied (Fig. 7G).

### ITF2357 attenuates myofibroblast formation via regulation of Id3

**Figure 8:**
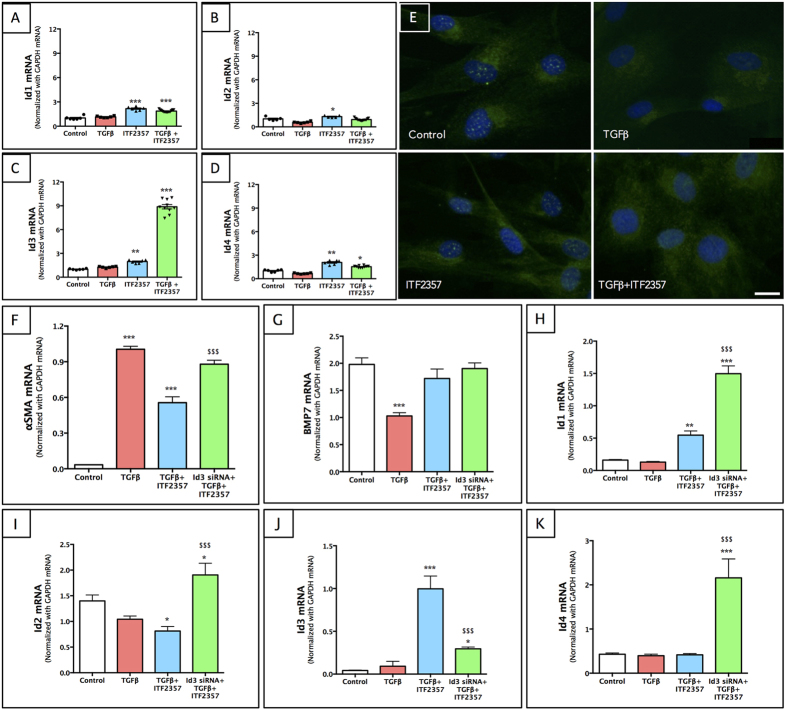
ITF2357 attenuates anti-fibrotic functions through Id3 regulation. (**A–D**) All Id proteins (Id1-4) transcripts were expressed in pHCSFs. ITF2357 alone induced a significant increase in the expression of (**A**) Id1, (**B**) Id2, (**C**) Id3 and (**D**) Id4. (**C**) Id3 mRNA showed a striking 10-fold increase in the presence of TGFβ + ITF2357. (**E**) Immunostaining of Id3 protein in pHCSFs showed decreased expression in TGFβ only treated group, but an increased expression in the ITF2357 treated groups regardless of TGFβ. Scale bar = 50 μm. (**F–K**) Subsequent Id3 knockdown using Lipofectamine transfection of Id3 siRNA showed up-regulation of (**F**) αSMA in Id3 siRNA + TGFβ + ITF2357 group to the level of that seen in TGFβ treated group only. (**G**) BMP7 mRNA production in Id3 knockdown remained unchanged. (**H**) Id1, (**I**) Id2 and (**K**) Id4 mRNA was upregulated in Id3 knockdown condition suggesting a compensatory mechanism. (**J**) Id3 siRNA transfection efficiency was found to be ~75%. *p < 0.05; **p < 0.01 and ***p < 0.001 against control. ^$$$^p < 0.001 against TGFβ + ITF2357. There were n = 6–10 in mRNA expression and n = 4 in immunostaining for each group. Error bars represent SEM.

To further evaluate the downstream targets of the BMP7 signaling pathway, we examined the regulation of inhibitors of differentiation (Id) proteins for their role in cellular differentiation and fibrosis. pHCSFs treated with ITF2357 showed increased expression of Id1-Id4 in a time-dependent manner (data not shown). Also, ITF2357 treatment to pHCSFs significantly increased transcript levels of Id1 (Fig. 8A, p < 0.001), Id2 (Fig. 8B, p < 0.05), Id3 (Fig. 8C, p < 0.01) and Id4 (Fig. 8D, p < 0.01). Interestingly, pHCSFs showed maximal expression of Id3 expression in the presence of TGFβ + ITF2357 (~10-fold) but minimal expression in Id1 (~2-fold), Id2 (no change) and Id4 (~1.5 fold) compared to the control group. Similarly, immunostaining of pHCSFs treated with ITF2357 showed a significant increase in the expression of Id3 protein in the presence or absence of TGFβ (Fig. 8E). A previous study by Kowanetz *et al.*^51^ proposed Id3 to play an important role in the epithelial-mesenchymal transitions (EMT) in lens epithelial cells. Therefore, we decided to further investigate the functional role of Id3 in the regulation of transdifferentiation of fibroblasts to myofibroblasts in the presence of ITF2357+/−TGFβ treatment.

To accomplish this, we used Id3 specific siRNA in an *in vitro* model of corneal fibrosis. pHCSFs were transfected with Id3 siRNA, treated with ITF2357 and/or TGFβ for evaluation of αSMA, BMP7 and Id’s genes (Fig. 8F–K). Transfection efficiency was found to be ~75% (Fig. 8J, p < 0.001 against TGFβ + ITF2357) as determined by Id3 transcript levels. Id3 knockdown resulted in an overall amplification of Id1 (Fig. 8H), Id2 (Fig. 8I) and Id4 (Fig. 8K) transcripts, which could be attributed to their compensatory mechanism. Interestingly, ITF2357 was unable to rescue the transdifferentiation of fibroblasts to myofibroblasts in the pHCSFs transfected with Id3 siRNA as measured by αSMA expression (Fig. 8F, p < 0.001 compared to the TGFβ + ITF2357 groups). BMP7 transcript levels remained unchanged in Id3 knockdown group (Fig. 8G).

## Discussion

The present study demonstrated safety, efficacy and mechanistic insights into the anti-fibrotic role of a histone deacetylase inhibitor, ITF2357 via regulation of Id3 involving TGFβ/BMP7 signaling pathways in an *in vitro* and *in vivo* model of corneal fibrosis. We found that ITF2357 at nanomolar concentrations (50–500 nM) was safe in primary cultures of human corneal fibroblasts (pHCSFs) with minimal influence on cell viability, proliferation and migration.

Most importantly, we found that ITF2357 inhibits corneal fibrosis at two levels – transdifferentiation of fibroblasts to myofibroblasts and ECM production, via regulation of TGFβ/Smad/BMP7 signaling pathways involving multiple steps. ITF2357 effectively antagonized αSMA production in pHCSFs stimulated with TGFβ *in vitro* and abrogated clinical haze formation in a photorefractive keratectomy (PRK)-treated *in vivo* rabbit model of corneal fibrosis. Although we observed similar recovery in corneal thickness in ITF2357- and MMC-treated PRK corneas, there were more viable stromal cells in ITF2357 group compared to MMC treated group. Pathway analysis in TGFβ-treated pHCSFs suggest a protective role of ITF2357 in cellular responses to TGFβ signaling via down-regulation of Smad2/3/4 complex and its responsive gene, P4HA1, involved in collagen production, while increasing BMP7 production and transcription of its downstream signaling targets, inhibitor of differentiation proteins- Id1, Id2, Id3 and Id4. Further molecular analysis established that transfection of pHCSFs with Id3-specific siRNA was unable to rescue TGFβ-driven transdifferentiation of fibroblasts to myofibroblasts in the presence of ITF2357. These results establish for the first time molecular mechanisms deciphering the role of ITF2357 in the inhibition of corneal fibrosis.

HDACi are newly described anti-cancer drugs that exerts their inhibitory role by modifying acetyl modifications from histone tails, increasing cellular hyperacetylation index, leading to cell cycle arrest, protein degradation, and eventually in tumor cell apoptosis^24^. ITF2357 has been tested in Phase II clinical trial for Hodgkin’s lymphoma, chronic myeloproliferative diseases and juvenile idiopathic arthritis, and is currently undergoing evaluation for long-term study on chronic myeloproliferative neoplasm (ClinicalTrials.gov Identifier: NCT01761968). In addition to its anti-tumor effects, ITF2357 has been shown to reduce cytokine levels involved in various inflammatory and autoimmune diseases^41^,^42^. This is the first investigation for its therapeutic role in fibrosis. Previously, first generation HDACi such as TSA, and its derivative- Vorinostat have been suggested to inhibit fibrosis^32^,^33^,^34^,^35^. However, unlike TSA (insoluble in water), ITF2357 is orally active and shows longer sustained hyperacetylation of histone proteins as compared to SAHA^36^. Moreover, TSA is only effective at μM concentrations and long-term safety data and downstream molecular targets or mechanisms are yet to be known.

Next, we looked into mechanism of action involving ITF2357 driven regulation of TGFβ/Smad/BMP7 signaling axis in corneal fibrosis. Since ITF2357 directly modulates transcriptional activity by hyperacetylation of histone proteins in pHCSF, we observed repression of TGFβRII along with Smads expression at the transcription level, and reduced phosphorylation of Smad2 and Smad3 protein, indicating the overall suppression in cellular responses to TGFβ stimulus. At the same time, since the role of myofibroblast is centred on ECM production in wound remodeling, significant reduction of P4HA1, which is downstream in the signaling pathway, further resonates sustained inhibitory effects of ITF2357 at several steps in TGFβ pathway.

On the other hand, TGFβ responses are intrinsically antagonized by a parallel pathway led by BMP7 for the regulation of myofibroblast differentiation^52^. BMP7 signaling is facilitated by phosphorylation of Smad1, Smad5 and Smad8, which competes with Smad2/3 in the TGFβ pathway to associate with co-activator Smad4 for nuclear translocation. Phosphorylation of Smad1/5/8 has been known to activate transcriptional machinery that opposes TGFβ responses^53^. This is supported by the recent reports where BMP7 gene transfer into the cornea has been shown to treat fibrosis in an animal model of cornea scarring^23^,^54^. In this study, we found that ITF2357 treatment to pHCSFs increased transcriptional machinery of BMP7 signaling components as observed by the increased expression of Smad5, Smad8 and BMP7 in the presence and/or absence of TGFβ stimulation. Interestingly, BMP7 levels were undetectable in the control or TGFβ-stimulated pHCSFs but showed a massive increase in the presence of ITF2357. This led us to investigate further downstream of BMP7 signaling pathway that might be facilitating its anti-fibrotic effects.

Inhibitors of differentiation (Id) genes are evolutionarily conserved BMP7-responsive transcriptional regulators involved in multiple cellular processes including cell proliferation, differentiation and ultimately, defining cell fate^55^. The members of Id family (Id1-Id4) share conserved sequence homology in the basic helix-loop-helix motif for binding to E proteins^56^ and prevents its binding to DNA for dominant-negative transcriptional inhibition. Moreover, Id proteins determine cellular responses to TGFβ and BMP7 signaling pathway. Of the 4 members in the family, Id1 and Id3 expression have been reported to be down-regulated in TGFβ-driven epithelial-mesenchymal transitions and up-regulated by BMP7 for the inhibition of cellular differentiation^51^,^57^. In human fetal lung fibroblasts, stimulation with TGFβ induced Id1 and Id3 expression, which drives phenotypic switching between the fibroblasts and myofibroblasts in smooth muscle cells^58^. This was also seen in prostate cancer, where Id1 and Id3 were induced by TGFβ to inhibit proliferation^59^. However, in epithelial cells, Id2 and Id3 were transcriptionally suppressed by TGFβ, in contrast to sustained induction by BMP7^51^. This could be explained by the differential cell-specific transient and dynamic expression of Id’s in response to stimulus, and preferential binding to different targets involved in diverse functions^55^. Nevertheless, in line with the anti-fibrotic role of BMP7, Izumi N^60^ showed decreased collagen I levels and tissue inhibitor of metalloproteinases-2 (TIMP2) via induction of Id2 and Id3 in pulmonary myofibroblasts. Therefore, we decided to investigate ITF2357-induced regulation of the Id family of proteins, which is downstream of TGFβ/BMP7 signaling pathway, if any. We found that all four members of Id proteins (Id1-Id4) were transiently expressed in pHCSFs. ITF2357 treatment in the presence or absence of TGFβ stimulus transcriptionally activated expression of Id1–4 with the maximum stimulation of Id3 at the transcript and protein levels. The most interesting finding was observed in the pHCSFs transfected with Id3-specific siRNA, which failed to rescue the cellular transdifferentiation in the presence of ITF2357 and TGFβ, as observed with the high levels of αSMA expression suggesting a vital role for Id3 in myofibroblast generation during corneal fibrosis.

In conclusion, we established a safety and efficacy profile for the topical application of HDACi, ITF2357 to inhibit corneal fibrosis in a PRK model of corneal wound healing. Molecular mechanisms revealed that ITF2357 regulates multiple steps in the TGFβ/BMP7 signaling pathways and its responsive gene, P4HA1. We also suggest that Id3 acts like a molecular switch to decide the cellular fate for fibroblast transdifferentiation in corneal fibrosis (Supplementary figure S5).

## Materials and Methods

### Primary Cultures of Human Corneal Stromal Fibroblast Cells (pHCSFs)

Human donor cornea-sclera rims were obtained from Miami Eye Bank (Miami, FL) and Saving Sight (Kansas City, MO). Central cornea was excised using an 8.0 mm trephine, and corneal epithelial and endothelial were removed by light scraping using a Beaver #64 blade. Remaining cornea stroma was cut into small 1–2 mm pieces and placed onto 35 mm Primaria Culture dishes (BD Biosciences, Franklin Lake, NJ). DMEM/F-12 with 10% FBS, 1X ITS, 1% pen/strep and 0.1% AmphoB was used to maintain explant cultures in 37 °C with 5% CO_2_ till confluence. Explant pieces were removed carefully using sterile forceps and pHCSFs were expanded up to 4 passages. Media with 5% FBS was used to maintain subsequent passages. Passage 2 pHCSFs were used for all *in vitro* experiments. Unless otherwise stated, cultures were maintained for 3 days to reach 80% confluence before drug treatments.

### *In vitro* drug treatment

ITF2357 (diethyl-[6-(4-hydroxycarbamoyl-phenyl carbamoyloxymethyl)-naphthalen-2-yl methyl]-ammonium chloride; monohydrate; Givinostat Italfarmaco, patent WO 97/43251, US 6034096) was a generous gift from Italfarmaco, Research Center, Cinisello Balsamo, Milan, Italy. To reconstitute, 1 mg of ITF2357 was added to 1 ml distilled water and heated to 90^o^ C until dissolved^36^. ITF2357 was kept at room temperature according to the manufacturer recommendations. In addition, 0.02% ITF2357 was prepared for *in vivo* topical application. Concentrations of 50 nM to 5000 nM were initially tested in several *in vitro* experiments. Dilutions were made in pHCSFs media without FBS. Subsequently, 250 nM was used as a standard dose for the entire *in vitro* study. To examine the effects of HDACi on corneal fibrosis, pHCSFs were treated with ITF2357, in the absence or presence of 1 ng/ml TGFβ1 for 72 hr. TGFβ1 was used to induce fibroblast to myofibroblast transdifferentiation in pHCSFs^5^. All pHCSFs treatments were done in serum free conditions.

### Ethics statement

The Institutional Animal Care and Use Committee of SingHealth, Singapore approved all the animal protocols used in this study. All procedures in animals were done in accordance to the guidelines of the Association for Research in Vision and Ophthalmology (ARVO) Statement for the Use of Animals in Ophthalmic and Vision Research.

### Animals and Photorefractive Keratectomy (PRK) surgery

Twelve to fifteen week old New Zealand White rabbits (2.5–3.5 kg) were obtained from National University of Singapore. Animals were anesthetized intramuscularly with 50 mg/kg ketamine hydrochloride (Parnell Laboratories, Alexandria, Australia) and 5 mg/kg xylazine hydrochloride (Troy Laboratories, Smithfield, Australia). Photorefractive Keratectomy (PRK) of −9.0 diopter was performed as describer earlier^6^ with minor modifications. In brief, a 9 mm diameter area in the central cornea was first marked out using a trephine, and epithelium within area is scraped off using a Beaver #64 blade (BD Biosciences). PRK with 6 mm ablation zone on the central stroma was performed using Technolas 217z excimer laser (Bausch & Lomb Surgical, Munchen, Germany). A metallic mesh was positioned in the path of excimer laser after 50% of excimer laser pulses were fired to induce surface irregularities on the corneal tissue. One eye of each rabbit underwent the procedure while the contralateral eye served as naïve control. All rabbits were followed for 4 wk and euthanised by overdose intravenous injection of sodium pentobarbital.

### *In vivo* application of Mitomycin C (MMC) or ITF2357

The rabbits were divided into 4 groups with 6 eyes each: (I) Naïve control, (II) PRK control, (III) PRK + ITF2357, (IV) PRK + MMC. In PRK control, 0.2 ml of balanced salt solution (BSS) was instilled onto the eye for 2 min, then drained off. In ITF2357 treatment, 0.2 ml of 0.02% ITF2357 was instilled onto the eye for 10 min immediately following PRK, then drained off. The eyelids of the rabbit were lifted to form a cup to hold the drug solution for maximum contact. The treatment was repeated for 4 consecutive days at the same time each day. In MMC-treated group, sponges soaked with 0.02% MMC were placed directly onto the area of ablation for 2 min immediately after PRK. The eye was washed copiously with BSS after application. MMC was obtained from Singapore National Eye Centre (SNEC, Singapore) pharmacy and was only applied once. 0.3% Tobrex® antibiotic ointment was applied onto every PRK eye for 4 days (Alcon Laboratories, Inc., Puurs, Belgium) to prevent infection. All groups were clinically examined using slit lamp and *in vivo* confocal microscopy every week for up to one month.

### Biomicroscopic grading of corneal haze

The severity of corneal opacity/corneal haze was assessed weekly for 4 weeks by slit lamp microscope (Nikon FS-3 V; NIKON, Tokyo, Japan) according to classification system reported previously^48^.

### *In vivo* confocal biomicroscopy

*In vivo* confocal biomicroscopy was performed postoperatively at 1, 2, 3 and 4 wk using a scanning confocal microscope (HRT3; Heidelberg Engineering GmbH, Heidelberg, Germany). An immersion fluid, carbomer gel (Vidisic Mann Pharma, Berlin, Germany) was used to prevent direct contact between the corneal surface and the objective lens. Central cornea of each rabbit was examined with 2 z-axis scans from the epithelium to the endothelium, and vice versa. Semi-quantitative analysis of the level of reflectivity from three images with the brightest reflectivity in the anterior stromal layer were done by measuring the mean gray value of the reflective particles using ImageJ software (Wayne Rasband, National Institutes of Health, Bethesda, MD).

### Anterior segment optical coherence tomography (ASOCT) biomicroscopy

Pre- and post-PRK rabbit corneas underwent weekly ASOCT examination (RTVue, Optovue, Inc., Fremont, CA) for up to one month. Central corneal thickness was measured using RTVue software according to the manufacturer recommendations.

### Rabbit cornea collection

After euthanasia, corneas were excised and cut into halves. One half was embedded in OCT cryo-compound (Leica Microsystems, Nussloch, Germany), and cut into 8 μm thick sections using cryostat (Microm HM 550; Microm, Walldorf, Germany), for immunofluorescence staining. The other half was flash frozen in liquid nitrogen and stored at −80 °C for mRNA and protein analyses.

### Immunofluorescence

Immunostaining in pHCSFs was performed in 8-well culture slides (BD Biosciences) pHCSFs were fixed with 4% paraformaldehyde (Sigma-Aldrich, St. Louis, MO) for 10 min, and washed twice with 1X phosphate buffered saline (PBS). Cells were permeabilised with 0.15% Triton X-100 for 15 min and blocked with 5% BSA + 0.1% Triton X-100 for 30 min. This was then followed by the addition of primary antibodies for 90 min, and secondary antibody for 60 min at room temperature. Slides were mounted with Vectashield containing DAPI (Vector Laboratories Inc., Burlingame, CA), and imaged with Zeiss Axioplan 2 fluorescence microscope (Zeiss, Oberkochen, Germany). Quantification of positive stained cells was done in three non-overlapping anterior stromal fields from central cornea. For tissue immunohistochemistry, 8 μm sections of rabbit corneas were fixed with 4% paraformaldehyde (Sigma) for 10 min and processed for immunostaining as mentioned above.

### Real-time Polymerase Chain Reaction

Total RNA was isolated and purified from cornea tissue using RNeasy Mini Kit according to manufacturer’s instructions (Qiagen Inc., Valencia, CA). To obtain RNA from pHCSFs, culture dishes were first washed twice with sterile 1X PBS, and scrapped with a rubber policeman in lysis buffer. Reverse transcription for synthesis of cDNA was performed using Superscript III (Life Technologies). 500 ng of RNA was used as a starting material. Real time PCR was performed on the LightCycler 480 II System (Roche, Indianapolis, IN) using SYBR green dye (Thermo Scientific, Waltham, MA). The cycle profile was pre-incubation at 95 °C for 5 min, 55 amplification cycles at 95 °C for 10 s, 60 °C for 15 s and 72 °C for 30 s. Melting curve analysis was done to verify amplification reaction. HPRT was used as the housekeeping gene. Cp values of the amplifications were taken for relative quantification against naïve control. Primers used are illustrated in Table 1.

### Western Blot

Cornea tissue and pHCSFs were lysed in RIPA buffer with protease and phosphatase inhibitors (Sigma). Extracts were sonicated, centrifuged and supernatant was quantified using BCA protein assay kit (Pierce, Thermo Scientific). 15 μg of protein was separated on 4–20% pre-cast gels (Bio-Rad, Hercules, CA, USA) for 30 min at 200 V. Transfer onto PVDF membrane was done overnight at 40 V at 4 °C. The blots were blocked in 5% non-fat dry milk (Sigma) dissolved in TBST for 1 hr, followed by primary antibody in blocking buffer for 1 hr at room temperature. After three washes for 10 min each with TBST, blots were incubated with HRP-conjugated secondary antibody and visualized using SuperSignal Chemiluminescence (ThermoFisher Scientific). Protein expression quantification was normalized to GAPDH as housekeeping standard.

### xCELLigence System

The xCELLigence system (ACEA Biosciences, San Diego, CA) was used to investigate pHCSFs migration, proliferation and cell death analysis after ITF2357 treatment. We used E-plate 96 incorporated with sensor arrays at the bottom of each well, which detects and translates cell attachment in the form of electronic impedance. A parameter termed cell index (CI) was used for the purpose of calculations, which corresponds to the relative density and adherence strength of cells in each well. Approximately, 5000 pHCSFs were seeded per well. ITF2357 concentrations of 100 nM, 250 nM, 500 nM, 1000 nM, 2500 nM and 5000 nM were tested. Cells were maintained for 72 hr in the xCELLigence RTCA SP Station, placed in an incubator at 37 °C with 5% CO_2_. Data from ITF2357 groups were normalized to control group without ITF2357.

### PrestoBlue Assay

pHCSFs were seeded at 5000 cells per well on a flat-bottom 96 well plate (Greiner, Monroe, NC, USA). ITF2357 in varying concentrations (50 nM, 100 nM, 250 nM, 500 nM, 1000 nM, 2500 nM, 5000 nM) was added 24 hr after seeding. Cell viability was measured at 4, 8, 24, 48 and 72 hr using PrestoBlue Cell Viability Reagent (Life Technologies). This cell permeable resazurin-based colorimetric method is a cell viability indicator that measures the reducing power of living cells to quantitatively estimate the proliferation of cells. Absorbance is measured using microplate reader (Tecan, Zanker Road, San Jose, CA) at 570 nm, with reference values at 600 nm.

### TUNEL assay

To determine extend of apoptosis, pHCSFs were seeded in 8-well culture slides and treated with 50 nM, 100 nM, 250 nM, 500 nM, 1000 nM of ITF2357 for 72 hr. Apoptotic cell death was detected by terminal deoxynucleotidyl transferase dUTP nick end labeling (TUNEL) assay using *In Situ* Cell Death Detection Kit (Roche) according to manufacturer’s instructions. Cell nuclei were stained and mounted with Vectashield containing DAPI and viewed under Zeiss Axioplan 2 (Zeiss) fluorescence microscope.

### Scratch wound assay

pHCSFs were grown on a 6-well plate to 100% confluency and scrapped with a sterile 1 ml micropipette to create a uniform scratch wound. Plates were washed lightly with sterile 1X PBS to remove cell debris. Cells were treated with 250 nM, 500 nM, 1000 nM, 2500 nM and 5000 nM concentrations of ITF2357 and monitored for cell migration under light microscope for 4 days. Microscopic pictures were taken every 6 hr for accurate measurement of wound width. Two parallel lines were drawn at the scratch boundaries by a single masked observer to demarcate wound width. ImageJ was used to measure wound width, and percent of wound closure was calculated by taking the fraction of remaining width at time-point over initial width at zero hr.

### Histone acetylation

To quantify levels of acetylated-lysine present on histone tails after ITF2357 treatment, pHCSFs cultured in T75 flasks were treated with varying concentrations of the drug (100–1000 nM). Cells were collected by trypsinisation with TrypLE Express (Gibco). Nuclear histones were isolated and purified using the EpiSeeker Histone Extraction Kit (Abcam, Cambridge, MA, USA). In brief, harvested cells were first centrifuged at 1000 rpm for 5 min at 4 °C. Supernatant was removed and cells were lysed in 1X pre-lysis buffer for 10 min on ice with gentle stirring on orbital shaker. Suspension was spun down at 3000 rpm and re-suspended in lysis buffer, followed by further 30 min incubation on ice without agitation. After centrifugation at 12,000 rpm for 5 min, supernatant containing acid-soluble proteins was transferred into fresh vial for protein quantification and western blot. Acetyl-lysine antibody from Millipore was used at 1:500 concentrations overnight at 4 °C, followed by secondary at 1:5000 for 1 hr at room temperature.

### Enzyme-Linked Immunosorbent Assay (ELISA) assay

To determine BMP7 levels, pHCSFs cultured on 60 mm Primaria plates (BD Biosciences) and conditioned media were collected 24 hr post treatment and spun down to remove debris. BMP7 levels were measured using the Human BMP7 Quantikine ELISA Kit (R&D Systems, Minneapolis, MN, USA) according to manufacturer’s instructions.

### Id3 siRNA transfection

To knockdown Id3, pHCSFs were transfected with specific siRNA (Forward: 5′AGAGUAUAUAGGUUUUGUAtt′3; Reverse: 5′UACAAAACCUAUAUACUCUat′3) using Lipofectamine RNAiMAX assay (Life Technologies). Silencer® Select Negative Control and BLOCK-iT™ Alexa Fluor® Red Fluorescent Control were used as negative and positive controls, respectively. pHCSFs were plated at high density to obtain 80% confluence in 24 hr. Cells were cultured and transfected in the absence of antibiotics and serum. siRNA and Lipofectamine reagents were prepared separately, and only incubated together for 10 min immediately prior to adding to pHCSFs. The final concentration of Id3 siRNA in culture was 60 nM. Cells were incubated with transfection compound for 8 hr, then washed with fresh serum-free media before immediate drug treatment with TGFβ and ITF2357.

### Statistical Analysis

Statistical analysis was performed using Student t-test for comparison between 2 groups. Post-hoc analysis between treated groups and control were performed using one-way ANOVA (Graphpad Prism 6.0, GraphPad Software, Inc., La Jolla, CA, USA). Significance was taken when p < 0.05. Cells with αSMA- or TUNEL-positive staining were counted in six randomly chosen field for each treatment. Percentage of staining was calculated by dividing number of positive stained cells with total number of cells in each field of view.

## Additional Information

**How to cite this article**: Lim, R. R. *et al.* ITF2357 transactivates Id3 and regulate TGFβ/BMP7 signaling pathways to attenuate corneal fibrosis. *Sci. Rep.* **6**, 20841; doi: 10.1038/srep20841 (2016).

## Supplementary Material

Supplementary Information

## Figures and Tables

**Table 1:** List of primers used in the real time PCR.

Gene	Accession No.	Forward Sequence (5′–3′)	Reverse Sequence (5′–3′)	Amplicon
αSMA	BC093052.1	ctgttccagccatccttcat	tcatgatgctgttgtaggtggt	70
BMP7	M60316	agcccgggtagcgcgtagag	gcgccggtggatgaagctcga	163
Collagen I	NM_000088.3	gggattccctggacctaaag	ggaacacctcgctctcca	63
Collagen IV	NM_001845.4	tggtgacaaaggacaagcag	ggttcaccctttggacctg	72
Fibronectin	NM_212478.1	ctggccgaaaatacattgtaaa	ccacagtcgggtcaggag	114
P4HA1	NM_001142595.1	tctcgtcccactttccagag	ggccaaggaaagaggatcac	89
Smad1	NM_005900.2	caggaagccctttccagat	cctgggtcatggggtctt	75
Smad4	NM_005359.5	cctgtggcttccacaagtc	ctgatgctatctgcaacagtcc	76
Smad5	NM_001001419.1	aggcgacatattggaaaagg	tgaggcattccgcatacac	67
Smad7	NM_005904.3	cgatggattttctcaaaccaa	aggggccagataattcgttc	86
Smad8	NM_001127217.2	tgccacagctgatagacatgtag	catagtaggcgaccgagcac	101
Id1	NM_181353.2	ctctacgacatgaacggctgt	tgctcaccttgcggttctg	76
Id2	NM_002166.4	tcagcctgcatcaccagaga	ctgcaaggacaggatgctgata	92
Id3	NM_002167.4	tcagcttagccaggtggaaatc	tggctcggccaggactac	76
Id4	NM_001546.3	ccgagccaggagcactagag	cttggaatgacgaatgaaaacg	116
