# Supplementary material for: ITF2357 transactivates Id3 and regulate TGFβ/BMP7 signaling pathways to attenuate corneal fibrosis
Source: Sci Rep. 2016 Feb 11;6:20841. doi: 10.1038/srep20841 (PMC4750002; doi:10.1038/srep20841)
Supplement: Supplementary Information [file srep20841-s1.pdf]

ITF2357 transactivates Id3 and regulate TGFβ/BMP7 signaling pathways to attenuate corneal fibrosis

Rayne R. Lim[1,2], Alison Tan[3,4], Yu-Chi Liu[3,5], Veluchamy A. Barathi[3,6,7], Rajiv R. Mohan[1,8,9], Jodhbir S. Mehta[3,4,5], Shyam S. Chaurasia[1,2,3,4*]

[1]Ocular Immunology and Angiogenesis Lab, Department of Veterinary Medicine & Surgery, University of Missouri, Columbia, MO 65211
[2]Department of Biomedical Sciences, University of Missouri, Columbia, MO 65211
[3]Singapore Eye Research Institute, 20 College Road,169856, Singapore
[4]Institute of Molecular and Cellular Biology, A*Star, Biopolis, Singapore
[5]Singapore National Eye Centre, 11 Third Hospital Avenue,168751, Singapore
[6]Ophthalmology and Visual Sciences Academic Clinical Program, Duke-NUS Graduate Medical School, 8 College Rd, 169857, Singapore
[7]Department of Ophthalmology, Yong Loo Lin School of Medicine, National University of Singapore, Singapore
[8]Harry S. Truman Memorial Veteran Hospital, Columbia, MO 65201, USA
[9]Mason Eye Institute, University of Missouri, Columbia, MO 65211, USA

[*]**Corresponding author:**

Shyam S. Chaurasia, MS, PhD
Assistant Professor in Ophthalmology and Vision Sciences
Ocular Immunology and Angiogenesis Lab
One Health One Medicine Ophthalmology & Vision Sciences Program
Department of Veterinary Medicine and Surgery
University of Missouri
Room E115A Vet Med Building
1600 East Rollins Street
Columbia, MO 65211-5110
Tel: (573) 882 3807
E-mail: chaurasias@missouri.edu

Legends to Supplementary Figures

Supplementary figure S1. ITF2357 did not affect cell migration at nanomolar concentrations (≤500nM). (A-F) Scratch wound assay performed on pHCSFs cells treated with ITF2357 showed no inhibitory effects on cell migration at 250nM up to 96 hr in culture. Scratch boundaries indicated by black parallel lines. Dotted lines indicate wound boundary at hr 0. (G) Wound width plotted as a percentage of initial scratch (at 0 h) over duration of experiment (96 hr). Scratch wounds in (A) control and (B) 250nM ITF2357 showed 80% closure after 96 hr, while (C) 500nM ITF2357 showed 60% closure. Wound closure in (D) 1000nM ITF2357 group plateaued after 48 hr, with significant retardation after 72 hr treatment. pHCSFs treated with (E) 2500nM and (F) 5000nM ITF2357 concentrations were not imaged following extensive cell death by 48 hr. ***, $p<0.001$ against control (at 96 hr time point). There were n=4 samples for each time point in every group studied.

Supplementary figure S2. ITF2357 hyperacetylates histone proteins. Nuclear histone protein extracts isolated from pHCSFs showed dose dependent increase in hyperacetylation of lysine residues with increasing concentrations (100nM-1000nM) of ITF2357. ***, $p<0.001$ against control. N=4 in each treatment. Error bars represent SEM.

Supplementary figure S3. Flow diagram showing *in vivo* rabbit experimental design. Twelve to fifteen wk old New Zealand white rabbits were divided into 4 groups:

(I) Control, (II) PRK (-9.0D), (III) PRK+ITF2357, (IV) PRK+MMC. 0.02% ITF2357 was given as an eye drop, and 0.02% MMC was applied onto cornea using a soaked sponge. Both drugs were administered immediately after laser treatment. Rabbits were imaged every week using slit lamp, specular and confocal biomicroscopy. At 4 wk, animals were euthanized and corneas were collected for biochemical analysis using immunohistochemistry and western blot.

Supplementary figure S4. ITF2357 did not affect central corneal thickness (CCT) after PRK. ASOCT images taken immediately after PRK and drug treatment showed damage to central cornea in (A) PRK (-9.0D) + ITF2357 (0.02%) and (B) PRK (-9.0D) + MMC (0.02%) rabbit corneas. Corneal tomography showed area of excimer ablation in the region of reduced thickness after (C) IT2357 or (D) MMC treatment. (E) CCT of rabbit corneas measured using RTVue software during clinical imaging did not reveal any differences in corneal recovery post-PRK for both the drug treatments. There were six rabbits in each group.

Supplementary figure S5. Schematic diagram depicting the molecular mechanism driving the action of ITF2357 on corneal fibrosis. ITF2357 drug applied topically onto cornea suppressed TGFβ signaling pathway, inhibiting phosphorylation of Smad2/3 and down-regulation of its co-activator Smad4. Furthermore, it also reduces TGFβ responsive gene-P4HA1 transcription, which led to the reduced maturation of pro-collagen fibrils, and hence overall decrease in ECM proteins in the cornea. In parallel, ITF2357 increases BMP7 and its signaling components Smad1/5/8. Most importantly,

ITF2357 transcriptionally activates Id3, a downstream target of the BMP7 pathway. Id3 acts like a molecular switch to decide the cellular fate between fibroblasts and myofibroblasts in corneal wound healing. Thus, ITF2357 regulate corneal fibrosis at two levels: (1) by inhibiting the transdifferentiation of fibroblasts to myofibroblasts and (2) by preventing the accumulation of irregular ECM matrix production.

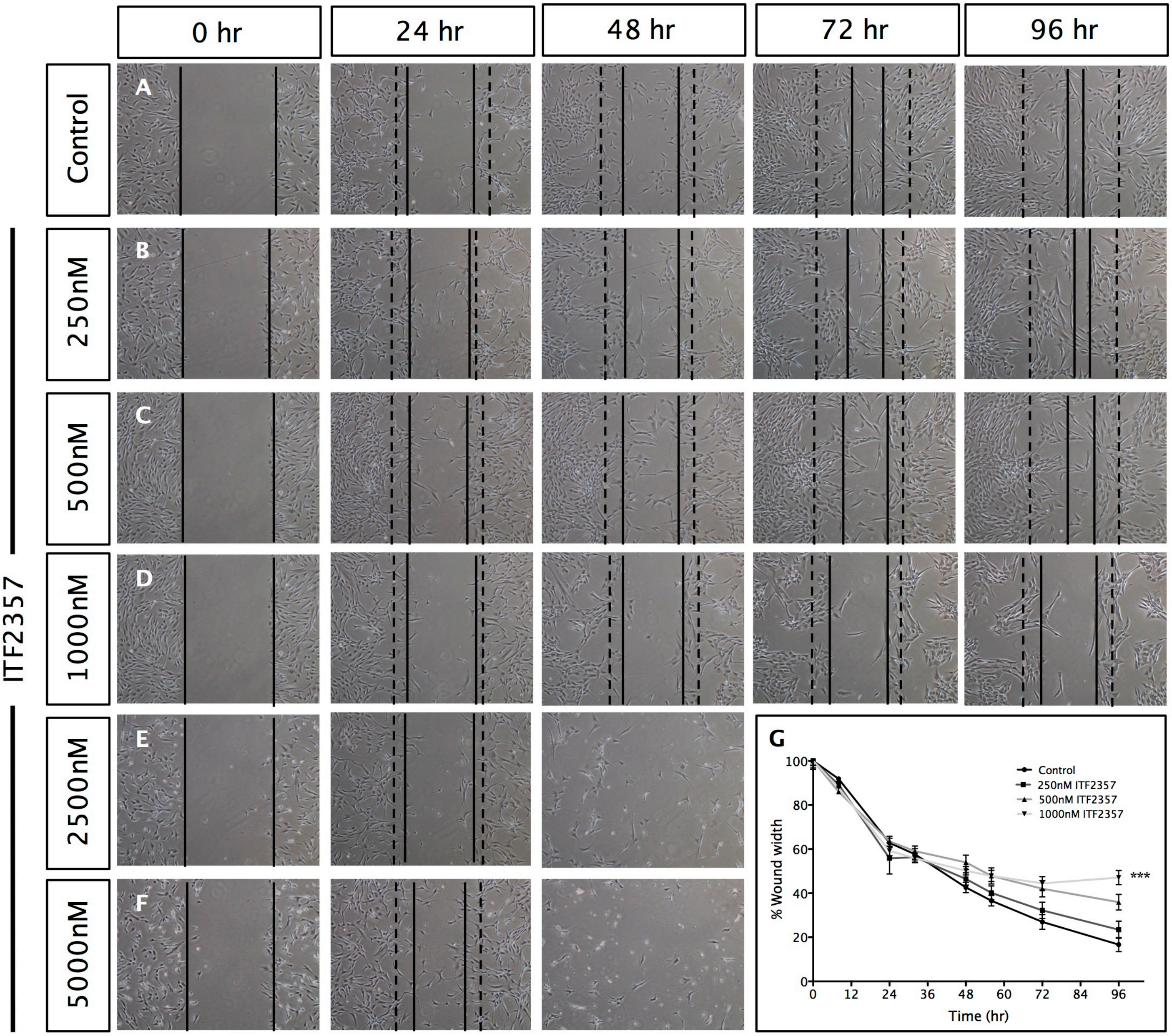

0 hr
24 hr
48 hr
72 hr
96 hr
Control
ITF2357
250nM
500nM
1000nM
2500nM
5000nM
A
B
C
D
E
F
G
Control
250nM ITF2357
500nM ITF2357
1000nM ITF2357
% Wound width
Time (hr)
***

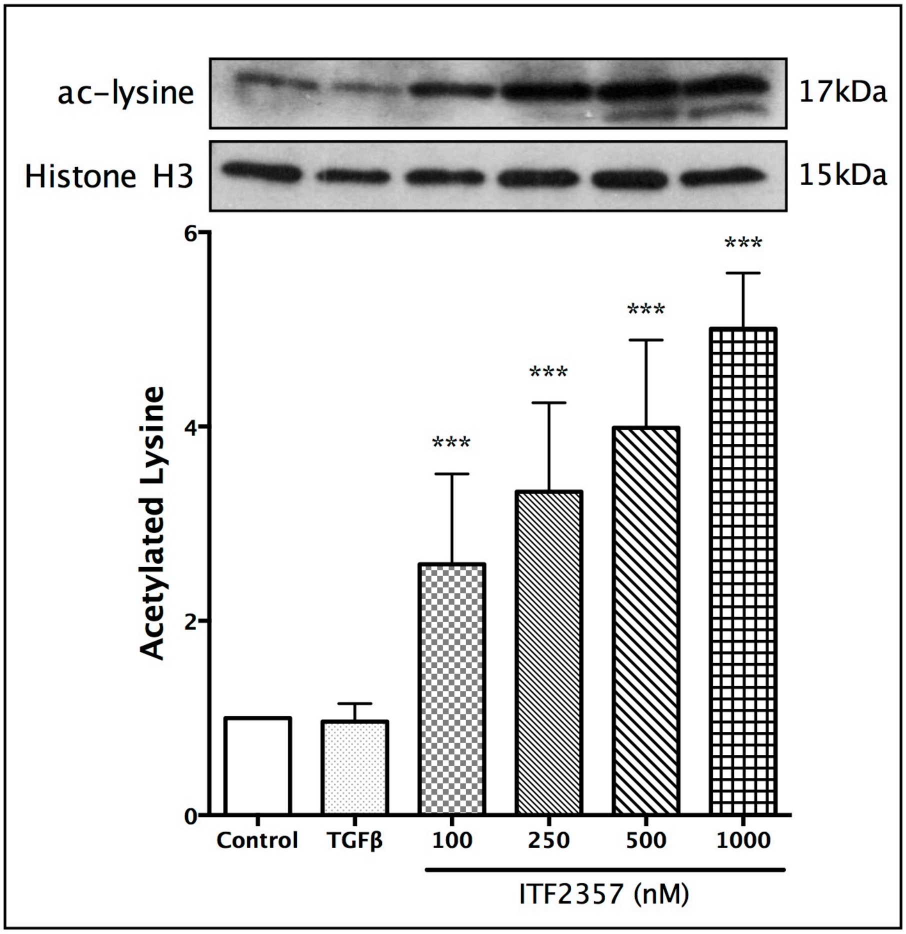

ac-lysine
17kDa
Histone H3
15kDa
Acetylated Lysine
0
2
4
6
***
***
***
***
Control
TGFβ
100
250
500
1000
ITF2357 (nM)

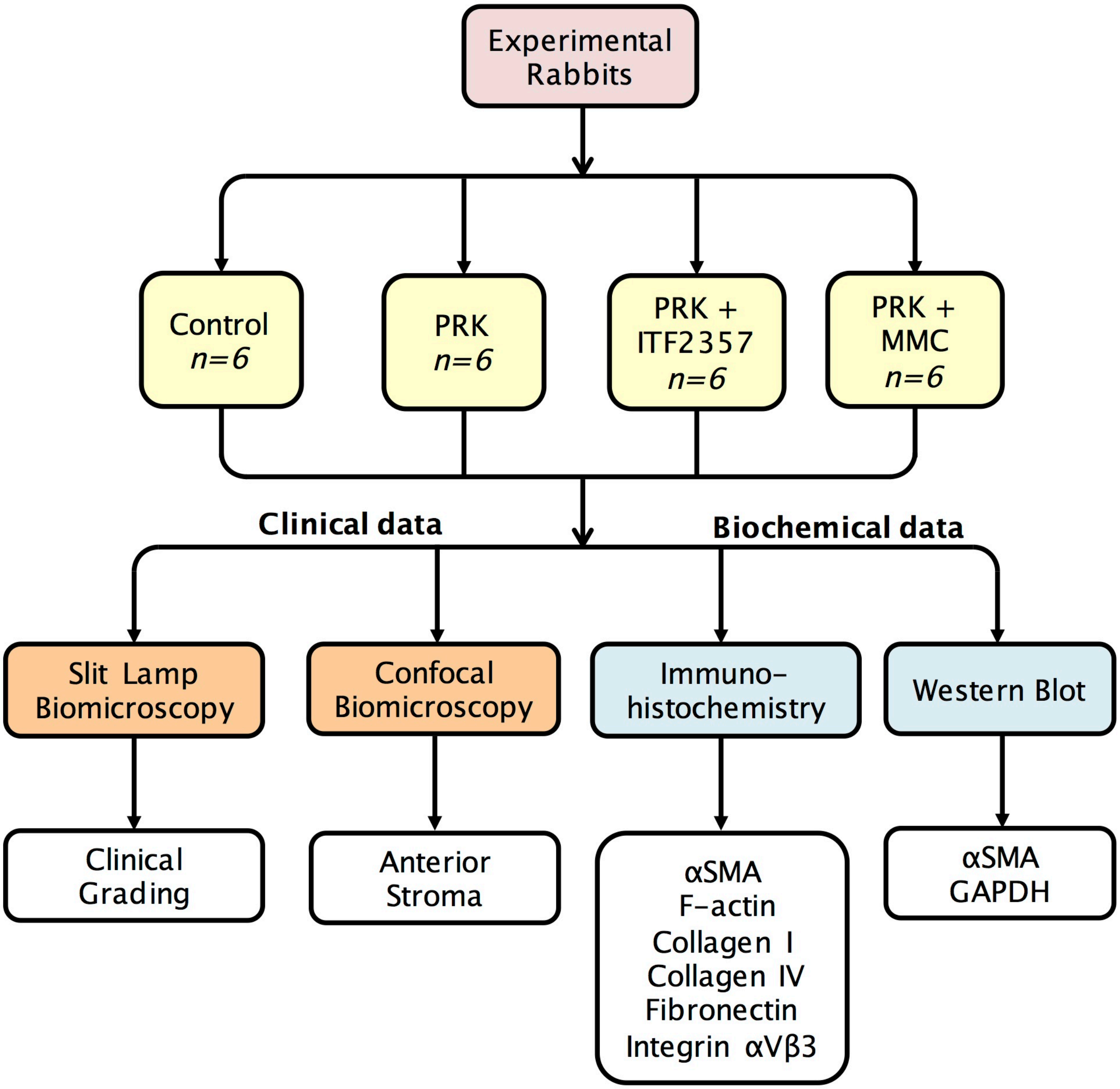
Experimental Rabbits
Control n=6
PRK n=6
PRK + ITF2357 n=6
PRK + MMC n=6
Clinical data
Biochemical data
Slit Lamp Biomicroscopy
Confocal Biomicroscopy
Immuno-histochemistry
Western Blot
Clinical Grading
Anterior Stroma
αSMA
F-actin
Collagen I
Collagen IV
Fibronectin
Integrin αVβ3
αSMA
GAPDH

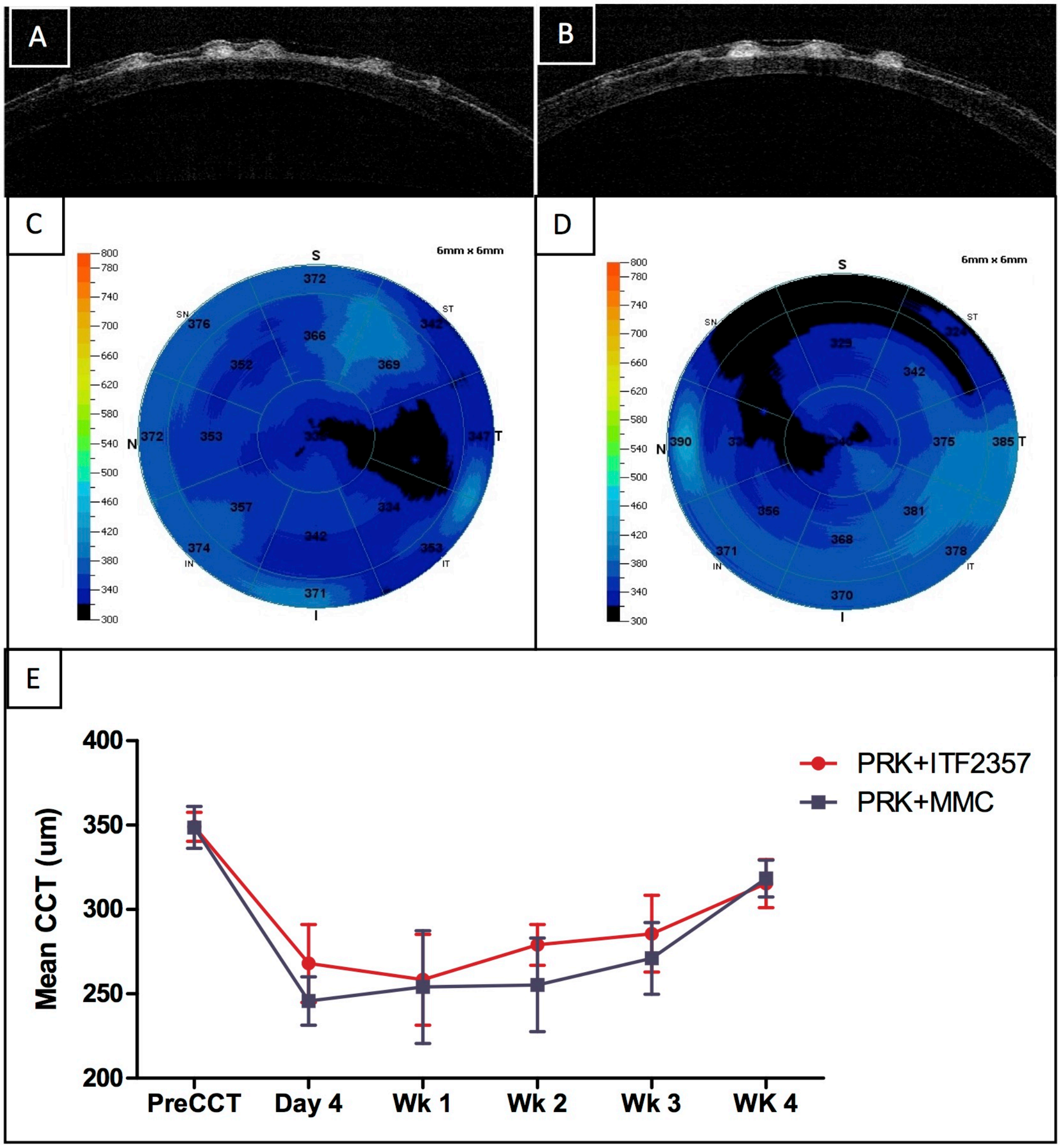

A
B
C
D
E
6mm x 6mm
S
N
T
I
SN
ST
IN
IT
Mean CCT (um)
PreCCT
Day 4
Wk 1
Wk 2
Wk 3
WK 4
PRK+ITF2357
PRK+MMC

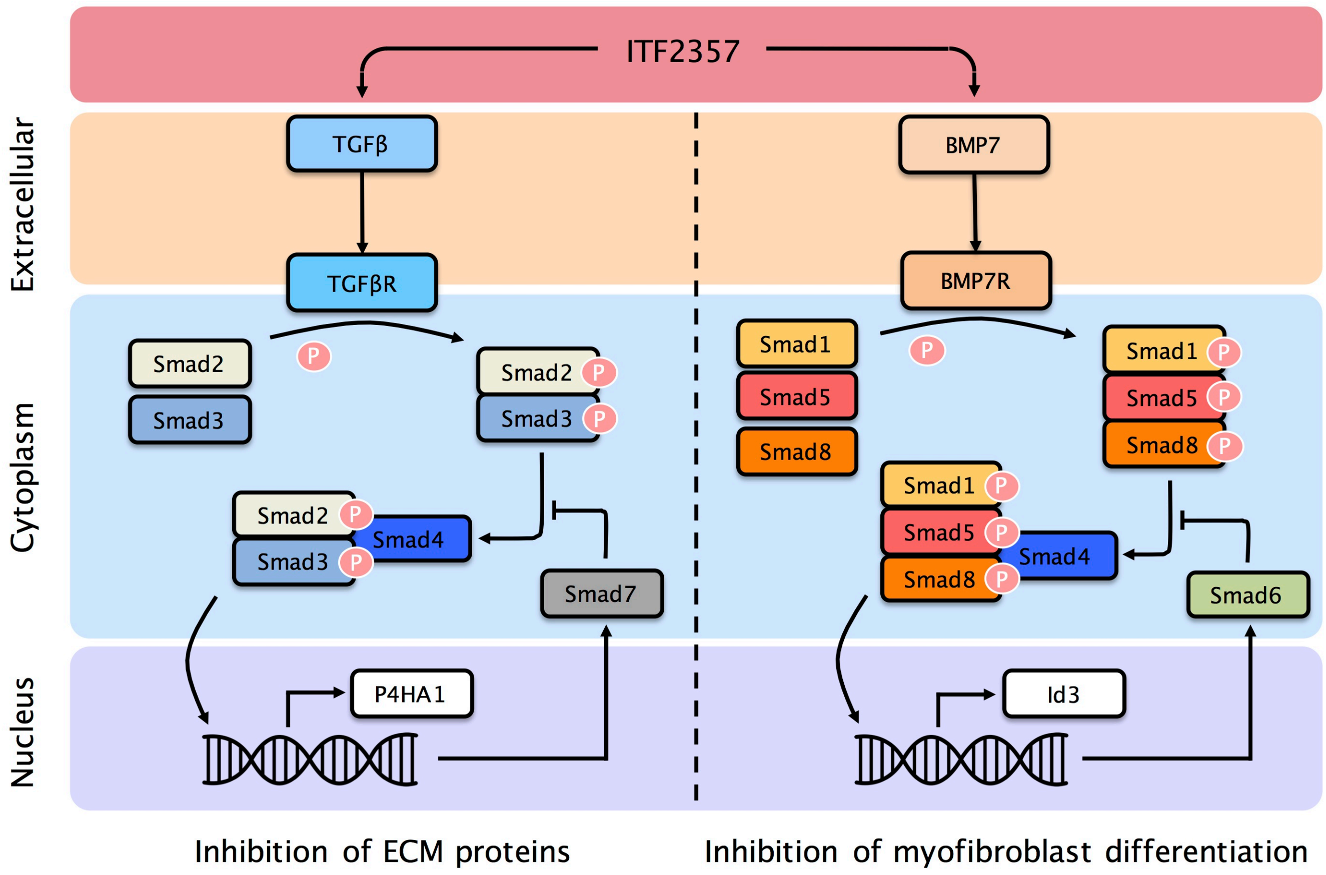
ITF2357
Extracellular
Cytoplasm
Nucleus
TGFβ
TGFβR
BMP7
BMP7R
Smad2
Smad3
P
Smad2
Smad3
Smad4
Smad7
P4HA1
Smad1
Smad5
Smad8
Smad6
Id3
Inhibition of ECM proteins
Inhibition of myofibroblast differentiation
